# New Perspectives about Relevant Natural Compounds for Current Dentistry Research

**DOI:** 10.3390/life14080951

**Published:** 2024-07-29

**Authors:** Stefania Dinu, Stefania-Irina Dumitrel, Roxana Buzatu, Dorin Cristian Dinu, Ramona Popovici, Camelia Szuhanek, Anamaria Matichescu

**Affiliations:** 1 Department of Pedodontics, Faculty of Dental Medicine, Victor Babes University of Medicine and Pharmacy, 9 No., Revolutiei 1989 Bv., 300041 Timisoara, Romania; dinu.stefania@umft.ro; 2 Pediatric Dentistry Research Center, Faculty of Dental Medicine, Victor Babes University of Medicine and Pharmacy, 9 No., Revolutiei 1989 Bv., 300041 Timisoara, Romania; 3 Department of Toxicology, Drug Industry, Management and Legislation, Faculty of Pharmacy, Victor Babes University of Medicine and Pharmacy, 2nd Eftimie Murgu Sq., 30004 Timisoara, Romania; dumitrelstefania@proton.me; 4 Department of Dental Aesthetics, Faculty of Dental Medicine, Victor Babes University of Medicine and Pharmacy, 9 No., Revolutiei 1989 Bv., 300041 Timisoara, Romania; 5 Family Dental Clinic, Private Practice, 24 Budapesta Street, 307160 Dumbravita, Romania; dorin@dr-dinu.com; 6 Department of Management, Legislation and Communication in Dentistry, Faculty of Dental Medicine, Victor Babes University of Medicine and Pharmacy, 9 No., Revolutiei 1989 Bv., 300041 Timisoara, Romania; ramona.popovici@umft.ro; 7 Department of Orthodontics, Faculty of Dental Medicine, Victor Babes University of Medicine and Pharmacy, 9 No., Revolutiei 1989 Bv., 300041 Timisoara, Romania; cameliaszuhanek@umft.ro; 8 Department of Preventive, Community Dentistry and Oral Health, Faculty of Dental Medicine, Victor Babes University of Medicine and Pharmacy, 14A Tudor Vladimirescu Ave., 300173 Timisoara, Romania; matichescu.anamaria@umft.ro; 9 Translational and Experimental Clinical Research Centre in Oral Health, Victor Babes University of Medicine and Pharmacy, 14A Tudor Vladimirescu Ave., 300173 Timisoara, Romania

**Keywords:** allicin, assay, curcumin, dentistry, eugenol, methods, natural compounds, oral health, rosmarinic acid, quercetin

## Abstract

Natural compounds have been used since the earliest civilizations and remain, to this day, a safer alternative for treating various dental problems. These present antimicrobial, anti-inflammatory, antioxidant, analgesic, and antimutagenic effects, making them useful in the prophylactic and curative treatment of various oral diseases such as infections, gingivitis, periodontitis, and even cancer. Due to the high incidence of unpleasant adverse reactions to synthetic compounds, natural products tend to gradually replace conventional treatment, as they can be just as potent and cause fewer, milder adverse effects. Researchers use several methods to measure the effectiveness and safety profile of these compounds, and employing standard techniques also contributes to progress across all medical disciplines.

## 1. Introduction

Since ancient times, dental care has been a concern for general health and well-being. Toothpaste was created thousands of years ago in Egypt, and it is the first known tool to be designed for dental care [1]. The evolution of medicine over the years has come to a great extent, starting from oral care products that contained mixtures of alum, salt, and vinegar to creating life-changing therapeutic agents, devices, and experimental methods that continuously improve the medical field [2,3]. Dentistry is one of the medical branches that is in constant development, with more and more researchers trying to improve and increase the efficacy of medical treatments and enhance the overall quality of life for patients [4]. Current research is focused on exploring natural compounds, mainly those derived from plants, to minimize as many local side effects as possible, such as allergic responses, inflammation, and systemic complications [5]. For this reason, natural compounds are sometimes chosen over synthetic ones, because they present fewer and milder negative outcomes, and can be just as potent, even in several types of oral cancer [6]. Among these plant-based bioactive compounds that present great interest in dentistry due to their antimicrobial, anti-inflammatory, antioxidant, and antitumoral properties are curcumin, quercetin, allicin, rosmarinic acid, and eugenol. These compounds proved positive effects on a variety of dental problems, from dental caries and periodontal diseases to oral cancer [7].

This article aims to analyze the current research on these natural compounds in the dentistry field, by examining their chemical properties, spectrum of action, the mechanism underlying their beneficial effects, and clinical performance. Moreover, methods and assays are described, because they are key factors, not only in assessing their efficiency and safety but also in providing a comprehensive overview of their potential in modern dental practice.

## 2. Natural Compounds Used in Dentistry

### 2.1. Curcumin (CUR)

#### 2.1.1. History

Curcumin is a compound present in Curcuma longa, also known as “turmeric” or “Indian saffron”, which belongs to the Zingiberaceae family. It has a long history of use that dates back thousands of years to traditional Chinese and Ayurvedic medicines in Indian culture. The flavor and color, along with its religious significance, made it a popular choice for cooking. Traditional herbal medicine has used it for digestive problems, anorexia, hepatoprotection, neuroprotection, diabetes, urinary tract infections, menstrual regulation, cancer, and as an overall immunomodulator that strengthens the body and heals the soul [8,9,10,11,12].

#### 2.1.2. Chemical Structure

**Figure 1:**
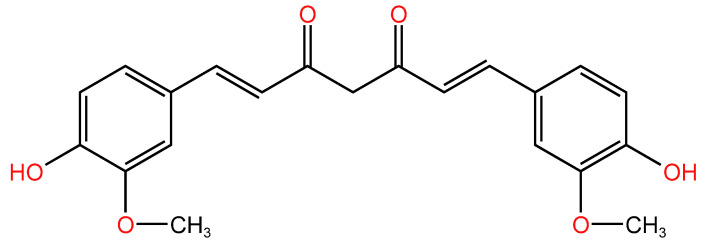
Molecular structure of curcumin.

Curcumin (Figure 1), also known as diferuloylmethane, is a symmetrical molecule, and it is the primary phytochemical of Curcuma longa [13]. It is the main curcuminoid compound that gives turmeric its bright yellow color. It is also a key element responsible for its antioxidant activity. It exhibits an antioxidant effect by donating electrons to oxygen reactive species (ROS), because of the presence of o-methoxiphenol group and methylenic hydrogen [14]. In general, curcumin is separated through solvent extraction [9]. 

#### 2.1.3. Antimicrobial Spectrum and Mechanism of Action

Curcumin presents a broad spectrum of action against gram-positive, and gram-negative bacteria, viruses, fungi, and parasites [15,16].

One of the mechanisms underlying the antibacterial effect is its action on the quorum sensing (QS) system. QS is a complex process that affects bacterial activity via intercellular communication and gene regulation. Bacteria send out signaling molecules called selfinducers, which can sense their population density. When the number of selfinducers rises, the bacterial population reaches a certain density, and gene expression adjusting begins. Curcumin inhibits QS and prevents bacterial adhesion, which is a critical step in biofilm formation, while also destroying the biofilm’s structure. The modulation of the QS system is also responsible for the antiviral effect [17].

#### 2.1.4. Formulations and Current Use of Curcumin in Dentistry

Curcumin has limited bioavailability and stability because of its hydrophobic nature, which represents a challenge when it comes to using it as a therapeutic agent. To overcome these problems, various approaches have been employed, such as innovative delivery systems, including nanoemulsions, nanomicelles, liposomal encapsulation, and colloidal nanoparticles, along with cyclodextrin inclusion complexes. Additionally, the use of polysorbates, phospholipid complexes, spray drying methods, and solid dispersions was effective in expanding the applications of curcumin in various products. Curcumin was included in mouthwashes (clinically tested), toothpaste (commercially available), gel (commercially available), chewing gum (clinically tested), lozenges (commercially available), and sprays (commercially available) [18,19,20,21,22,23,24]. Another issue encountered when formulating curcumin products is its yellow pigment, which can cause teeth and oral tissue staining, especially with prolonged use. Several strategies have been employed to mitigate this effect. For example, using tetrahydrocurcumin, one of curcumin’s main metabolites that lacks the α, β-unsaturated carbonyl group and does not have the yellow color, can eliminate the risk of staining. Alternatively, nano-curcumin formulations or chitosan-based products can be used to prevent staining [25,26,27,28]. One study reported that out of 30 subjects who used 0.1% turmeric mouthwash, none had teeth stains, and only 3 showed temporary tongue staining [29]. Other studies reported no teeth or mouth staining even after several applications of curcumin. However, more clinical studies conducted over longer periods are necessary to determine curcumin’s potential to stain buccal tissue and teeth as well as the longevity of this side effect [30,31,32].

It is well known to have many benefits against a large number of pathologies because it is a potent anti-inflammatory, antioxidant, antimicrobial, anticoagulant, and antimutagenic compound. Curcumin can successfully address issues related to dental pain because of its analgesic properties and ability to reduce swelling [15]. It can also serve as a natural alternative to CHX mouthwash in the treatment of gingivitis since it reduces plaque index and helps with gingival bleeding with fewer adverse effects [33]. In addition, the antioxidant, antimutagenic, and immunostimulant properties make curcumin a remarkable landmark in treating periodontal diseases and oral cancers [15]. Due to its anti-inflammatory properties, curcumin is effective in treating oral lichen planus, which is a chronic inflammatory disease that can significantly impact patients’ quality of life, resulting in dry mouth that leads to speech difficulties, ulcerative lesions, and problems with masticating certain foods, and it can sometimes affect sleep quality [7,34,35]. Evidence supporting curcumin usage is presented in Table 1.

#### 2.1.5. Adverse Effects

Curcumin is well-tolerated and considered generally safe for dental use, although it can sometimes cause contact dermatitis. If swallowed, it can result in gastrointestinal problems such as irritation, nausea, and diarrhea. Additionally, there have been some reports of tongue staining due to the high concentration of yellow pigment, which easily adheres to the surface. However, the staining was temporary, disappearing with brushing [15,36,42,43,44].

#### 2.1.6. Experimental Methods Used to Evaluate the Efficacy and Safety of CUR in Dental Applications

The cytotoxic effect of CUR on human PDL (periodontal ligament fibroblasts) was tested using MTT assay. The viability of the cells was measured 48 h post-treatment with concentrations of 25, 50, and 100% curcumin. No cytotoxic effects were observed at any of these doses [45]. Sukumaran et al. compared the cell viability of gingival fibroblast cells after treatment with different concentrations of curcumin and chlorhexidine (CHX) at various time points, using the MTT assay. After 24 h, the MTT assay revealed that CUR at 0.003, 0.03, 0.06, 0.1, and 0.12% over time periods of 1, 2, 4, 6, 8, and 10 min had a concentration- and time-dependent effect on the cell viability. CUR showed the best results, decreasing cell viability at the lowest concentration (0.003%) from 99.04 to 68.6% over the 1–10 min period compared to CHX, which decreased fibroblast viability at the lowest concentration (0.03%) from 77.99 to 53.62% at the same time intervals. Overall, curcumin exhibited less cytotoxicity at each concentration at any given time point compared to CHX [46]. Another study investigated curcumin’s effect on the viability of human gingival fibroblasts (HGFs). The MTT assay revealed after 24 h that curcumin at concentrations of 0.1–20 µM did not affect cell viability, whereas concentrations of 30 and 50 µM significantly induced cytotoxic effects on HGF cells in a dose-dependent manner [47]. The cytotoxic effects of curcumin were tested using the MTT assay on dental pulp stem cells (DPSC). After 24, 48, and 72 h, the MTT assay showed a significant increase in cell viability post-treatment with CUR at 0.5 and 1 µM. However, the viability of DPSC cells was significantly decreased at higher concentrations of 5, 10, and 15 µM after 1, 2, 3, and 7 days [48].

Curcumin (0.05 mg/mL) loaded nanoparticles (NPs) were tested in vivo for their effectiveness in treating mouse-induced periodontitis. The animals were killed after one and two weeks to observe the outcomes. µCT analysis demonstrated that curcumin-loaded NPs enhance bone formation after 14 days. Additionally, an increase in collagen content in the gingival tissue was also seen after 14 days, as shown by the analysis of the picrosirus red-stained sections. Immunohistochemical analyses were performed using the biotin-streptadivin-HRP-DAB method and revealed that the Runx2 protein expression (crucial for osteoblast differentiation) increased after 2 weeks. These results prove that curcumin loaded-NPs are beneficial for repairing gingival tissue and bone healing in mouse-induced periodontitis [49]. 

A study was conducted on participants suffering from oral submucous fibrosis (OSF) to test the efficacy of a 1 mg curcumin extract combined with coconut oil on OSF-related symptoms, such as interincisal mouth opening (IIMO) problems, pain, and burning sensation. IIMO was measured using Vernier calipers, and the pain and burning sensations were quantified using the visual analog scale. After a twice-daily application during a 3-month trial, it was concluded that the curcumin extract is effective in alleviating the side effects, making curcumin a possible adjuvant treatment in patients with OSF [50]. Ramezani et al. investigated the potential role of curcumin in alleviating radiotherapy-induced oral mucositis (OM) in a trial involving 37 patients with head and neck cancers. The subjects were divided into three groups: Group 1 received a curcumin mouthwash (0.1% *w*/*v*), Group 2 a soft gel containing 40 mg of curcuminoids as nano-micelles, and Group 3 a placebo mouthwash. Treatments were administered for 1 min three times daily. Both curcumin groups presented a time-dependent reduction in pain and burning. At the end of the trial, the CUR mouthwash group presented the best results, with more than 33% of patients being ulcer-free compared to the CUR gel group, where 15% remained ulcer-free, and all patients in the placebo group still had OM [51]. Malekzadeh et al. tested the effects of nano-curcumin capsules on gingival inflammation in patients with gingivitis and mild periodontitis. In the study, 25 subjects received nano-CUR soft gel capsules (80 mg), while 23 subjects received placebo. The treatment was administered once daily after breakfast for 28 days and the results were examined on Days 0, 7, 14, and 28. The papillary bleeding index and modified gingival index were measured using William’s probe. At the end of the trial, the curcumin group experienced a time-dependent improvement in both parameters, with the most significant results observed after 2 and 4 weeks of treatment [28]. A recent study was conducted on 44 patients to analyze the effect of CUR on alleviating pain after root canal treatment for acute pulpitis in mandibular molars. One group (22 patients) received 400 mg CUR + 20 mg pepper one hour prior to the procedure, while the other group of 22 patients received placebo. The visual analogue scale was used to measure the pain score immediately after the root canal treatment and after 8, 12, 24, and 48 h post-operative. A significant decrease in pain score was observed in the CUR group in a time-dependent manner after 8, 12, and 24 h, with no need for emergency analgesics by the end of the trial, while 2 patients in the placebo group required analgesics [52]. A clinical trial was performed to compare the effects of nano-curcumin capsules with prednisolone on oral lichen planus (OLP). In the study, 57 patients were divided into two groups: Group 1 (29 subjects) received 80 mg of curcumin in nano-micellar soft gel capsules, and Group 2 (28 subjects) received 10 mg of prednisolone. Treatments were taken once daily for 1 month. The results were examined using the visual analogue scale after 1, 2, and 4 weeks of treatment. All patients presented a noticeable decrease in pain and burning sensations after 2 and 4 weeks, with no significant differences between the groups. These findings suggest that curcumin has potential as an adjuvant treatment for OLP, offering a safer alternative with better tolerability than oral corticosteroids (prednisolone) [53]. Another study investigated the effects of curcumin on patients with OLP to determine its efficacy in managing acute pain. They were divided into two equal groups: one group received a mucoadhesive paste containing curcumin, and the other was treated with local corticoids (0.1% betamethasone lotion and nystatin suspension three times daily to prevent candidiasis). The CUR group applied the paste 3 times daily for 12 weeks. The lesion size and pain score were examined after 1, 2, 4, 8, and 12 weeks. At the end of the trial, the size of the lesions decreased in both groups, with no significant difference between them. By the eighth week, six patients exhibited excellent results, with their erosive lesions transitioning to reticular forms. Patients from both groups did not complain about adverse effects [54].

### 2.2. Quercetin (QRC)

#### 2.2.1. History

The “Quercetin” name was established in 1857, and it comes from Quercetum (oak forest) [55]. It has been part of human diets for centuries since it is found in fruits (orange, apple, bananas, berries), vegetables (onions, potatoes, eggplant), nuts, plants (capers, rocket, dill), and beverages (tea, wine) [56,57].

#### 2.2.2. Chemical Structure

**Figure 2:**
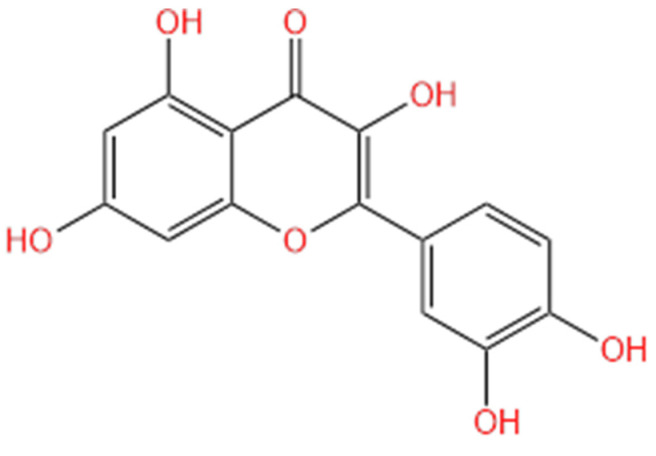
Molecular structure of quercetin.

The antioxidant activity of quercetin (Figure 2) is linked to the presence of a double bond between C2, C3, and the carbonyl group at C4 and the phenolic hydroxyl group [58].

#### 2.2.3. Antimicrobial Spectrum and Mechanism of Action

It acts against both gram-positive and gram-negative bacteria, and on some drug-resistant strains. It also has effects against fungi and viruses [59].

The presence of hydroxyl groups contributes to quercetin’s solubility and antibacterial effects by facilitating the compound’s interaction with the bacterial cell. Its permeability facilitates passage through the membrane and its subsequent destruction, leading to the leakage of cytoplasmic components. Moreover, it prevents biofilm formation because it interferes with the quorum sensing system. Another mechanism underlying the antibacterial effect is its inhibitory effect on the synthesis of nucleic acid [59]. 

The antifungal effect is related to several processes such as the disruption of the plasma membrane because of the excess release of ROS. The ROS can further interfere with lipids, which are crucial components of the fungal cell wall. Other mechanisms involved are thought to be due to inhibition of protein and nucleic acid synthesis, cell proliferation, and cell wall formation [60].

The antiviral activity involves binding viral capsid proteins, preventing the virus from attaching and entering host cells, as well as inhibiting DNA gyrase and blocking essential viral enzymes [59].

#### 2.2.4. Formulations and Current Use of Quercetin in Dentistry

Because of its limited solubility in water and poor bioavailability, the use of quercetin in various formulations for the oral cavity is still under research. Nanoemulgel has shown promising results in vitro for periodontitis treatment. Additionally, lipid-carrier-loaded nanocomposite presented positive effects when applied prior to implant placement to promote osteointegration. A study suggested that a cellulose-composited hydrogel containing QRC was suitable for antibacterial topical applications. These delivery systems have increased quercetin’s bioavailability and demonstrated good physical stability and sustained release of the drug. A QRC-containing mouthwash was tested in vitro and was effective in treating various pathogen-related oral infections. The formulation of a quercetin gel showed promising results in the treatment of minor aphthae. However, their clinical efficacy needs to be established in further studies in vivo. In the dentistry field, quercetin can be found in topical cream [61,62,63,64,65,66,67].

Especially after the SARS-CoV-2 pandemic, quercetin was brought into discussions for its multiple beneficial actions in improving the severity of COVID-19 disease by reducing the incidence and length of hospitalization, the need for non-invasive oxygen therapy, the progression to intensive care units, and the mortality rate. Additionally, it is well tolerated by patients and demonstrates anti-fatigue and appetite-stimulating properties. An unhealthy diet and a stressful lifestyle harm dental health. In the worst cases, when the diseases evolve, patients start losing teeth. Dental implants would be the solution to this issue. Unfortunately, this medical procedure comes with many complications: infections, insufficient osseointegration, or inflammation such as peri-implantitis. Studies focus on the potential of quercetin as an adjuvant treatment after medical interventions to prevent and treat any complications. Quercetin showed promising results in peri-implantitis management because of its anti-inflammatory and antibacterial effects as well as its role in the osteogenesis process. Treatment for peri-implantitis must address both inflammation and bone reformation, as gum swelling can lead to deterioration of the bone supporting the teeth. Quercetin actively targets a broad spectrum of both gram-positive and gram-negative bacteria, particularly those present in the buccal mucosa. It is effective against bacteria that do not respond to any other medical compounds. Moreover, by killing bacteria, QRC can reduce the deposition of bacterial plaque and tartar on the teeth, which can further lead to other dental problems such as caries, gingivitis, and periodontitis. Phytoestrogens, found in plants containing quercetin, significantly influence osteogenesis. Therefore, quercetin can serve as a remineralization agent [68,69,70,71,72]. Evidence supporting quercetin usage is presented in Table 2.

#### 2.2.5. Adverse Effects

Systemic administration of QRC via intravenous injection at concentrations above 1400 mg/m^2^ can induce emesis, breathing difficulties, and nephrotoxicity (the safe dose was determined to be 945 mg/m^2^) [75]. In the dentistry field, however, quercetin is considered a generally safe and well-tolerated treatment for several problems. Several clinical studies have reported either no notable adverse effects or none at all. To fully quantify its safety in oral care, more human studies are necessary [66,67,70,75,76,77]. 

#### 2.2.6. Experimental Methods Used to Evaluate the Efficacy and Safety of QRC in Dental Applications

In an in vitro study, the effects of quercetin on oxidative stress induced by hydrogen peroxide (H_2_O_2_) in human gingival fibroblast cells (HGF) were examined. Using xCELLigence system it was observed that a concentration of 15 µM had the best results, increasing cell proliferation and viability by 21% after 6 h and by 41% after 48 h. A concentration of 20 µM initially induced a slight cytotoxicity in the first 6 h, but this effect was reversed after 48 h, showing a 22% increase in effectiveness. Confocal laser microscopy images, following green fluorescence staining, revealed that 15 µM QRC significantly reduced ROS production in H_2_O_2_-treated cells after 30 min of incubation. Additionally, qRT-PCR analysis showed that QRC upregulates the expression of type I collagen genes after being exposed to ROS for 3 h at a 15 µM concentration. Furthermore, the mitochondrial respiratory capacity was measured by the XF 24 Extracellular Flux Analyzer, and it was proven that after 30 min of treatment, QRC at a concentration of 15 µM enhanced mitochondrial respiratory capacity and stimulated more mitochondrial respiration compared to untreated cells. These results conclude QRC’s positive effects in reducing oxidative stress in HGF cells, increasing cell viability, and improving their metabolism and function [78].

Quercetin’s capacity to protect the dentin from erosion was tested on extracted human wisdom teeth using contact profilometry and the microhardness tester. Additionally, to observe the surface morphology, a scanning electron microscope was used. After these tests were completed, it was determined that QRC at 0.300 mg/mL can effectively protect the dentin against erosion [79].

In a study conducted by Huang et al., quercetin’s ability to ameliorate wound healing impairment and inflammation caused by advanced glycation end products secreted in diabetes mellitus was tested in relation to disease-related periodontitis. The MTT assay revealed that a concentration above 20 µM is beneficial in HGF-induced cell inflammation. The wound healing assay showed that QRC can heal tissue after 48 h at 5, 10 µM. ROS levels were measured using flow cytometry and were seen to be suppressed at concentrations of 5, and 10 µM in a dose-dependent manner. ELISA analysis demonstrated that QRC at 5, 10, and 20 µM effectively suppressed IL-6, and IL-8 levels, the action increasing with the concentration. Western blot analysis was used to determine the levels of cellular senescence (p16 is the marker) and pro-inflammatory proteins related to the NF-κB (Iκβ, p-Iκβ, p65 p-p65) signaling pathway. After the results, it was concluded that QRC has a dose-dependent action against p16 at concentrations of 5, and 10 µM and on the pro-inflammatory proteins at 5, 10, and 20 µM. These results attest to the beneficial effects of QRC on gingival inflammation and the wound-healing process [80]. 

Sulastri et al. investigated quercetin’s effect as a pre- and post-treatment for bacterial-induced periodontitis in 18 male Sprague Dawley rats. The rats were divided into 3 equal groups: Group 1 (control) received only the vehicle (CMC-Na 0.5%), Group 2 (Qp) was treated with quercetin suspension at 45 mg/kg/day one week prior to the induction of periodontitis and continued treatment for two weeks post-induction, and Group 3 (Qc) was treated with quercetin suspension at 45 mg/kg/day one week after the induction of periodontitis and continued treatment for two weeks post-induction. The dosage of quercetin used was equivalent to 500 mg in humans. Periodontal pocket depth (PPD) was assessed using the Michigan-O probe with Williams marks at the mesial and lingual sites of the rat’s incisor tooth. The inflammation score was measured by staining and examining the results under a microscope to observe the density of fibro-collagen formed in the tissue during the healing process. Both quercetin-treated groups showed a reduction in PPD scores and an increase in wound-healing activity compared to the control group. The Qp group exhibited the most significant improvement, suggesting quercetin’s potential protective effect during periodontitis conditions [81]. A recent study investigated the effect of quercetin on preventing oxidative-stress-induced injury of periodontal ligament cells and alveolar bone loss in periodontitis using 36 male C57BL/6 mice (8 weeks old). The mice were divided into three equal groups: control (group 1), periodontitis (group 2), and periodontitis treated with quercetin (group 3). Quercetin was administered at a concentration of 50 mg/kg on the first day after surgery, and results were examined after 10 days. Immunofluorescence staining at the end of the trial indicated that QRC increased NRF2 expression and reduced oxidative stress by decreasing the MDA levels and increasing the SOD levels in serum. RT-qPCR analysis revealed that QRC upregulated the mRNA expression of NRF2-mediated genes (HO-1, NQO-1, GPx3, and CAT). Furthermore, micro-CT analysis demonstrated that quercetin significantly decreased alveolar bone resorption in the buccal and palatal sites of the maxillary 2nd molars compared to the control group, with QRC proving to be safe at the end of the 10 days. H&E staining showed that QRC was safe throughout the 10-day trial, with no noticeable alterations in several histological segments (heart, liver, kidney, spleen, stomach) [82]. Another study demonstrated that QRC has the ability to decrease alveolar bone loss in 16 male Wistar rats at doses of 75 mg/kg/day and 150 mg/kg/day after 15 days of treatment by inhibiting MMP-8, increasing osteoblast activity, and decreasing osteoclast activity. Significantly better results were observed at 150 mg/kg/day [83]. Mooney et al. proved QRC’s positive effect on maintaining oral health in 10- to 12-week-old male C57B2/6J mice. Each experiment included 10 mice in the QRC treatment group and 5 mice in the control group. QRC was administered orally at a concentration of 40 mg/kg twice daily, starting 6 days before and continuing for one week after the induction of ligature-induced periodontitis. At the end of the study, the experimental group presented decreased gingival inflammation, lower levels of cytokines (TNF and IL-6), improved oral dysbiosis by increasing the number of commensal bacteria (*S. sanguinis* and *S. parasanguinis*), and reduced inflammation in periodontal tissue by increasing A20 expression to levels observed in healthy tissue [70]. 

Hamdy et al. tested a topical quercetin cream on 44 male patients to investigate its potential for treating minor aphthous ulcers. The subjects were divided into two equal groups: group 1 received a control treatment (benzydamine hydrochloride mouthwash), and group 2 used two or three dabs of the quercetin cream. Both treatments were applied 3 times daily for 10 days. The QRC group showed superior results, with a reduction in ulcer size and pain in 35% of the patients after 2 days, 90% after 4–7 days, and 100% by the end of the trial. Additionally, 90% of patients reported good tolerability in terms of taste and no irritation. In contrast, no patient in the control group showed signs of healing after 7 days, and only 60% presented clinical success by day 10 [67]. Another study compared the effect of QRC to benzydamine hydrochloride in the treatment of minor aphthae; 20 patients applied 2% QRC gel, while another 20 used 0.15% benzydamine hydrochloride mouthwash. Both treatments were used 3 times daily for 7 days. At the end of the trial, the QRC gel group showed the best results, with complete healing in 60% of patients and partial healing in 40%, compared to the benzydamine hydrochloride group, where 35% healed completely and 65% partially. Both groups experienced a decrease in pain scores in a time-dependent manner, with the QRC group presenting the best results, although the difference was not statistically significant. Furthermore, the QRC group reported no adverse effects during the treatment [66].

### 2.3. Allicin

#### 2.3.1. History

Allicin is a sulfur compound that was first isolated in 1944. Since then, its antibacterial properties have been documented, although it has been used for centuries since it is one of the main components found in garlic (*Allium sativum* L.) [84]. Garlic has a long history of use that dates back to the formation of the first civilizations. Hippocrates referred to garlic as a superfood with medicinal properties. It was one of the most important ingredients in the culinary and medical fields. It was used for wound healing; as a painkiller; for cardiovascular problems and respiratory problems; for blood sugar regulation, infections, gynecological disorders, diabetes; and more [85].

#### 2.3.2. Chemical Structure

**Figure 3:**
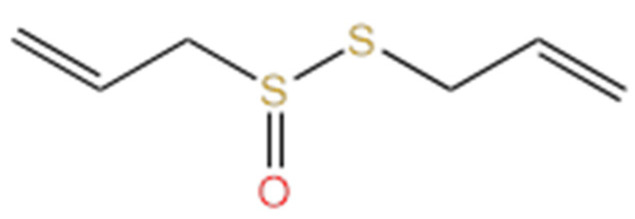
Molecular structure of allicin.

Allicin (Figure 3) is an alliin precursor, the main thiosulfinate found in garlic, and is responsible for garlic’s specific odor. Due to the presence of sulfur groups, and the oxidizing effect, it can react with thiols (-SH) from proteins, leading to various biological effects [86].

#### 2.3.3. Antimicrobial Spectrum and Mechanism of Action

Allicin acts against both gram-negative and gram-positive bacteria, multidrug-resistant strains, fungi, viruses, and parasites [87]. As mentioned earlier, its chemical structure allows it to interact with thiol groups from proteins and glutathione from bacteria and fungi. Allicin inhibits glutathione and disrupts the redox balance, causing oxidative stress that damages the cellular components, impairs cell cycle progression, and ultimately leads to cell death [86,88,89]. The mechanism underlying the antiviral activity of allicin occurs by disrupting the viral envelope and cell membrane. Because it interacts with thiol groups, it inhibits RNA polymerase, thereby preventing viral genome synthesis [90,91].

#### 2.3.4. Formulations and Current Use of Allicin in Dentistry

Allicin has reduced solubility in water, which makes it difficult to integrate it into several products. However, its inclusion in liposomes improved stability in water-based formulations, preventing precipitate formation for up to 50 days when stored at 4 °C. Allicin inclusion with α-cyclodextrin is another method of protecting it from degrading and also increases its stability in the aqueous phase, resulting in better bioavailability [92,93]. Most allicin research focuses on oral supplements and garlic extracts. However, in some clinical studies, it was included in mouthrinse, injection, and tablets. The insufficient number of in vivo studies on allicin formulations creates a gap in understanding its utility in various forms [94,95,96,97,98,99,100,101,102].

Allicin inhibits both caries-associated bacteria (*Streptococcus* spp.) and periodontitis-associated pathogens (*P. gingivalis*, *A. actinomycetcomitans*, and *F. nucleatum*). Due to its exceptional ability to combat *E. faecalis*, a common pathogen often encountered in root canal infections, it can serve as an effective root canal irrigator in endodontic treatment [84,87]. Because of its antioxidant and anti-inflammatory qualities, allicin can relieve toothache [103,104]. A study conducted on rats indicated that it enhanced the healing of gingival wounds by increasing the thickness of the epithelial layer [98]. It can also be a great candidate against oral candidiasis, as it inhibits *C. albicans* [105]. More recently, allicin’s potential benefit as an adjunctive treatment for stage II oral submucous fibrosis (OSF) was studied. OSF is a precancerous disease that creates high discomfort for patients since it causes ulceration, stiffness in mouth muscles, and burning sensations. Allicin has been shown to alleviate pain related to this disorder and improve overall oral health [99,106]. Supported evidence of allicin usage is presented in Table 3.

#### 2.3.5. Adverse Effects

Allicin use in dentistry is considered to have minimal to no side effects, however, there have been a few case reports of oral mucosal burn after its utilization [108]. It can also cause gastrointestinal irritation and increased bleeding [109]. The main problem with using allicin is its unpleasant odor and taste [93].

#### 2.3.6. Experimental Methods Used to Evaluate the Efficacy and Safety of Allicin in Dental Applications

Allicin’s antibacterial activity against *P. gingivalis*, and its inhibitory effect on the osteoclastogenesis process in osteoblast and osteoclast precursors were tested. The antibacterial activity was established after MIC, MBC, and time-kill curves were measured. The results revealed a MIC of 4 mg/L, an MBC of 8 mg/mL, and a time–kill curve similar to 0.05% CHX when it reached the MBC concentration. The concentration of 4 mg/mL reduced biofilm formation by half, while a concentration of 16 mg/mL reduced it by 90%. Confocal laser scanning microscopy recorded the biofilm reduction, with a concentration of 32 mg/mL effectively decreasing both the thickness of the biofilm and the number of living bacteria. The ELISA assay demonstrated that allicin could reduce the numbers of certain cytokines (M-CSF, RANKL) in a dose-dependent manner, which are responsible for better control of osteoclastic activity. The TRAP staining method assessed the low levels of cytokines, indicating a decrease in osteoclast formation. These methods serve as an excellent tool for identifying allicin’s potential benefits against periodontitis [110]. Pati et al. tested the effectiveness and safety of allicin in periodontitis by targeting the PD-1 pathway and reversing T-cell dysfunction. An in silico docking analysis demonstrated the interaction between allicin and PD-L1, confirming allicin’s high affinity and possible inhibitory effect on PD-L1 after calculating the binding energy. The MTT assay revealed that allicin decreased cell viability and induced cytotoxicity in T cells at the highest concentrations of 100 and 250 µM after 48 h. However, when the cells were treated with lower concentrations of allicin (0.5, 1, and 2.5 µM), there was an increase in the metabolic activity of CD3 + T lymphocytes. No cytotoxic effects were observed at concentrations of 5, 10, 25, and 50 µM. Allicin restored T cell function by downregulating TIM-3 and LAG-3 expression in a dose- (5, 10, 25, 50 µM) and time-dependent (24, 48, 72, 96, 120 h) manner, as shown through RT-PCR analysis. The most significant results were observed at a concentration of 25 µM between 48 and 96 h, which was also determined to be a safe concentration based on the MTT assay. These results prove allicin’s beneficial role in periodontitis by mediating crucial factors that can trigger or exacerbate this disease [111].

In a study, the effect of allicin against *C. albicans* and *S. aureus*, pathogens related to dental stomatitis, was investigated. MIC, MBC, and MFC (minimal fungicidal concentration) were measured using the broth microdilution method, and the results presented an equal MIC (8 µg/mL) for both pathogens, a MBC of 8 µg/mL, and a MFC of 16 µg/mL. Biofilm eradication assay was conducted using half of the MIC. Crystal violet assay was performed to indicate allicin’s potential destructive action against the biofilm and revealed that a concentration of 4 µg/mL was sufficient to reduce 50% of the *C. albicans* and *S. aures* biofilms after 5 min. These results indicate that allicin is a useful tool against denture stomatitis due to its powerful antimicrobial effect [112].

A clinical trial was conducted to investigate allicin’s potential for treating recurrent aphthous ulceration. The 96 subjects who participated were divided into 2 equal groups and were administered either 5 mg allicin tablets or vehicle only (placebo) 4 times daily for 5 days. The size and pain level of ulcers were measured on Days 1, 2, 4, and 6. At the end of the trial, it was noticed that allicin had a time-dependent effect on reducing both ulcer size and pain score. Significant improvements were observed by Day 4, with a nearly 60% decrease in both ulcer size and pain score. By Day 6, the allicin group exhibited a 72.5% reduction in ulcer size and a 75.7% decrease in pain levels. Additionally, the subjects demonstrated good tolerability during the treatment [101]. Oral allicin was tested on 20 patients with mucocutaneous lesions from Behcet’s disease. The subjects received 1 tablet (20 mg allicin) for 12 weeks, and the results were examined after 4, 12, and 16 weeks. Because one patient did not comply and another had an allergic response, 18 patients completed the trial. A time-dependent effect was observed in reducing skin lesions, and by the end of the trial, they were almost completely healed. Additionally, it greatly increased GSH and SOD levels while significantly decreasing MDA and MPO, thereby attenuating oxidative stress. These results demonstrated that allicin has a beneficial role in mucocutaneous Behcet’s disease [102].

An *Allium sativum* L. (garlic) ethanolic extract was tested on thirty 10-week-old male Wistar rats for 7 days to assess its potential in the gingival wound healing process. The rats were divided into 5 groups of 6 rats each: Group 1: negative control; Group 2: 20% garlic ethanolic extract gel; Group 3: 40% garlic ethanolic extract gel; Group 4: 80% garlic ethanolic extract gel; V-positive control (benzydamine). Chemical identification revealed that the gel contained allicin and alliin (which converts to allicin). It also contained flavonoids at concentrations of 0.73%, 0.68%, and 3.67% in the 20% extract gel, 20% extract, and 100% garlic extract, respectively. Each group was subdivided into 2 subgroups of 3 rats, and the results were examined after the animals were decapitated after 5 and 7 days. The treatment was applied 3 times daily. After 5 days, it was observed that all garlic gel extract concentrations increased epithelial thickness. After 7 days, the 40% and 80% garlic extract gel groups showed a significant difference in epithelial thickness compared to the negative control. The number of new blood vessels and fibroblasts after the garlic ethanolic extract gel application did not significantly differ compared to the negative control. In conclusion, garlic accelerated the epithelization process in the wound healing process, as indicated by the increase in epithelial thickness of gingival wound tissues in Wistar rats. Active constituents, such as allicin and flavonoids, may be responsible for this positive effect. However, further clinical and in vivo studies focusing specifically on allicin may help develop a fuller picture of its potential ability to help with gingival wound healing [98]. Another study investigated allicin’s effect on 30 Sprague Dawley rats to evaluate its capacity for re-epithelialization during the healing of oral ulcers. The rats were divided into two groups of 15, and one group served as the control, while the other received one drop of allicin twice daily 2 days post-ulcer-induction. Between Days 3 and 12, there was a significant increase in epithelial thickness. By Day 6, the experimental group had completely closed the wound and showed the formation of new connective tissue papillae, in contrast to the control group. From Days 9 to 12, the epithelial thickness decreased, indicating that it had entered a stage of maturation and remodeling compared to the control, where the epithelium remained thickened [113]. 

### 2.4. Rosmarinic Acid (RA)

#### 2.4.1. History

Rosmarinic acid is a significant compound found in rosemary (Rosmarinus officinalis). It was used for cosmetic purposes in several topical products in ancient Egypt. In traditional medicine, it was used for its cardioprotective, antidiabetic, antirheumatic, diuretic, and neuroprotective effects as well as for its activity on several respiratory problems. During the Great London Plague, the plant was used as a “shield” against Yersinia pestis when people traveled through contaminated areas. During World War II, its burned leaves were used against germs in a mixture that also contained juniper berries. It was not until more recently, in 1958, that RA was isolated from Rosmarinus officinalis. Since then, its antimicrobial, antioxidant, anti-inflammatory, anti-tumoral, and many other beneficial properties have been under study because it remains a relevant compound to this day [114].

#### 2.4.2. Chemical Structure

**Figure 4:**
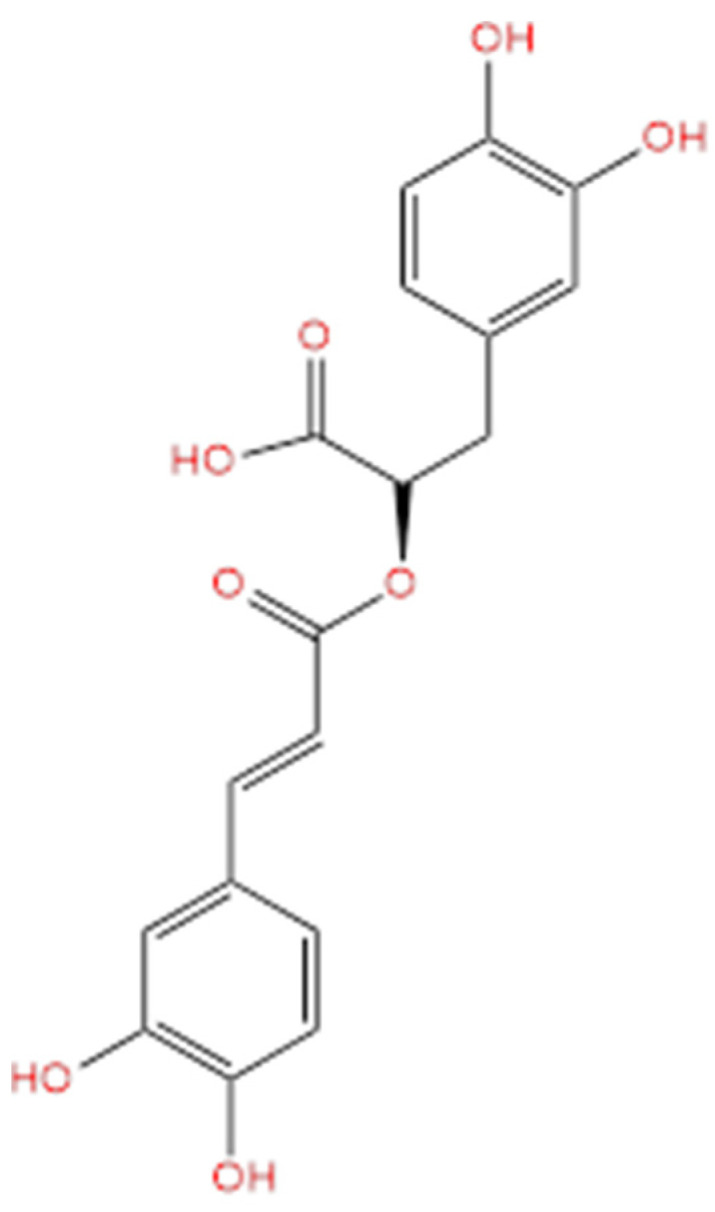
Molecular structure of rosmarinic acid.

RA (Figure 4) is a phenolic compound that has a structure formed from an ester of caffeic acid and 3,4-dihydroxyphenyllactic acid [115].

#### 2.4.3. Antimicrobial Spectrum and Mechanism of Action

Rosmarinic acid acts efficiently against a wide range of gram-positive, gram-negative, and multi-drug-resistant bacteria, as well as fungi [116,117]. Additionally, it presents antimicrobial activity against viruses and parasites [118,119]. 

The antibacterial effect hasn’t been fully elucidated, but it is thought to be related to the disruption of the bacterial cell membrane. It is believed to affect the membrane’s permeability, causing the intracytoplasmic components to leak and cause cell death [116,120]. The antifungal effect is also related to the alterations of the membrane’s permeability that lead to disruption in mitochondrial function [117].

Researchers have demonstrated the involvement of the hydroxyl moiety from the 3,4-dihydroxyphenyl group in the antiviral activity of certain strains. The hydroxyl moiety binds to a certain protein (EIII), blocking the active sites and preventing the host cell from binding [121].

#### 2.4.4. Formulations and Current Use of Rosmarinic Acid in Dentistry

Incorporating rosmarinic acid into various formulations while preserving its therapeutic properties can be quite a challenge. For this reason, addressing stability issues related to solubility and bioavailability is essential. Newer ways of delivering drugs, like liquisolid composites, solid lipid nanoparticles, chitosan-based nanoparticles made by spray drying, and loaded nanoemulsions appear to be promising options. These provided a sustained release of the compound as well as an increase in its stability and tolerability. However, further clinical studies are necessary to test these formulations in various oral pathologies. Before incorporating them into oral care products, it is essential to establish their efficacy in specific conditions [122,123,124,125].

Inflammatory biomarkers are key factors in maintaining good oral health because they are directly involved in several pathologies, as they can trigger tooth decay, gingivitis, and periodontal disease. RA was demonstrated to inhibit MMP-8, a metalloproteinase found in dentin. This inhibitory effect leads to a protective action on the dental structure, which can prevent dental caries and periodontal diseases [126,127]. Another benefit comes from its ability to reduce biofilm formation and gingival inflammation, which can further lead to other dental problems that can cause tooth loss, such as periodontitis [128]. Rosmarinic acid demonstrates potential as a prophylactic and curative treatment for oral cancer due to its antioxidant and anti-inflammatory properties. It regulates the cell cycle, reduces oxidative stress and chronic inflammation, prevents DNA damage, induces apoptosis, and inhibits metastasis in tumorous cells [129,130]. Supported evidence of rosmarinic acid usage is shown in Table 4.

#### 2.4.5. Adverse Effects

Rosmarinic acid is a well-tolerated compound with a low incidence of adverse effects. Several animal studies suggested RA’s safety in oral care for treating gingivitis, periodontitis, and oral carcinogenesis [133,134,135,136]. No severe negative outcomes were reported after oral use, but some patients complained about dry mouth and abdominal pain (if swallowed). However, more clinical studies are needed to assess its safety for oral care [137,138].

#### 2.4.6. Experimental Methods Used to Evaluate the Efficacy and Safety of RA in Dental Applications

In a study conducted by Othman et al., RA’s effect on the viability of dental pulp stem cells (DPSCs) was tested to establish its potential role as an intracanal medicament that can be used in regenerative endodontics. The MTT assay showed that after 3 days, RA can increase DPSCs viability at 6.25 and 12.5 µM, with no difference observed between the treated cells and the untreated ones at 25 and 50 µM. A decrease in cell viability was noticed at a 100 µM concentration. Additionally, after the MTT assay the density of the cells was quantified: At Day 3, no significant results were observed after treatment with 12.5, 25, and 50 µM. On Day 5, the concentrations of 12.5 and 25 µM significantly increased cell density, and on Day 7, only the 12.5 µM concentration significantly increased the density of the cells. The cell density and proliferation rate were assessed using a hemocytometer, and the results were examined under a light microscope. These findings indicate RA’s regenerative potential in endodontic treatment, as it enhances the viability of dental stem cells—which are vital for differentiating into other cell types, such as osteoblasts and odontoblasts—thereby maintaining the health of dental tissue [139].

Rosmarinic acid was tested at a concentration of 14 µg/mL on osteoblastic cells (MC3T3-osteoblastic cell line derived from mouse) on a titanium surface (a biomaterial used for dental implants) for 4, 7, and 10 days to establish its role in the cell’s differentiation and mineralization. The MTT assay showed that RA was able to improve the viability of MC3T3 on the titanium surface during differentiation after 4, 7, and 10 days. Through RT-PCR analysis, a rise in non-collagenous (ALP, BSP, DSPP, OCN) and collagenous (Col I) mRNA expression was observed in the osteoblastic cells after 4, 7, 10 days of treatment. The mineralization of the cells on the titanium surface was evaluated through ALP staining, and after MC3T3-E1 cell staining with Alizin Red S, formation of calcified nodules in a time-dependent manner was observed, a sign of bone formation. Additionally, RT-PCR analysis showed an increase in Runx-2 (at Day 7, the best results) and OPG (time-dependent effect) mRNA expression and a decrease in RANKL mRNA expression. After the western blot assay, a rise in the protein expression of Runx-2 was observed (Day 7 showed the best results) and OPG (time-dependent effect) and a decrease in the protein expression of RANKL. After measuring the mRNA and protein expression levels, the ratio of RANKL to OPG was observed to decrease. These methods were useful to finally conclude RA’s beneficial activity on osteoblastic cells on the titanium surface due to its osteogenic potential [137].

A *Salvia officinalis* L. (SO) extract, containing primarily rosmarinic acid along with luteolin derivatives, salvianolic acid F, and medioresinol, was tested on 14 12-week-old male Sprague Dawley rats in vivo to assess its potential role in new bone formation in the expanded premaxillary suture. The LC-MS/MS analysis confirmed that rosmarinic acid was the major component of the extract. The male rats were divided into two groups: Group 1 received the SO treatment, and Group 2 served as the control. The experimental group (Group 1) was administered a dose of 20 mg/kg/day via the orogastric route for 17 days. At the end of the study, the animals were euthanized and the results were examined. Compared to the control group, the SO group demonstrated significant new bone regeneration. This was evidenced by an increase in the newly formed bone area, a higher number of capillaries, and a greater intensity of the inflammatory cell response. Additionally, the SO group exhibited a significantly higher number of osteoblasts and osteoclasts than the control group. The effect of rosmarinic acid on osteogenesis has previously been demonstrated in the context of osteoporosis and the enhancement of osseointegration of implants on titanium surfaces. Therefore, its potential role in bone regeneration in this study is plausible. However, further clinical or in vivo studies focusing solely on rosmarinic acid are required to confirm its full potential for bone formation in the expanded premaxillary suture [133,137,140,141,142]. In a study, RA’s effect was tested on experimentally induced hamster buccal pouch carcinogenesis. RA (100 mg/kg) was orally administered 3 times a week for 14 days. After 2 weeks of treatment, it was observed that RA suppressed oral carcinogenesis by decreasing lipid peroxidation and improving antioxidant status. Additionally, it was noticed that rosmarinic acid down-regulated the expression of Bcl-2 and p53, thereby proving its anticancerous benefits [135]. Thymus satureioides (TS) extract was tested on Wistar rats with tongue-induced ulcers to analyze its potential effect on combating this disease. The animals were divided into 2 groups of 10 rats each, and gels containing TS extract at 5 and 10% were applied daily for 3 days. After 3 days, the 10% TS gel promoted complete healing of the tongue ulcer. Rosmarinic acid and salvianolic acid had the strongest binding affinity among the docked compounds on TLR-4, MMP-9, COX-2, and TNF-α. These results suggest that RA plays a substantial role in the healing process of tongue ulcers by reducing the expression of pro-inflammatory proteins, thereby inhibiting inflammation, apoptosis, and proteolysis [143]. In a study, the effects of rosmarinic acid on Sprague Dawley rats were analyzed for its potential in the treatment of oral ulcers. The trial included 60 rats that were divided into six equal groups: control, model group, 20 mg/kg RA group, 40 mg/kg RA group, 80 mg/kg RA group, and Watermelon Frost group. All rats received daily treatment for one week, except for the control group. At the end of the trial, the best outcomes were observed in the 40 mg/kg and 80 mg/kg RA groups and the Watermelon Frost group, with a significant reduction in ulcer area and IL-18, IL-1β, and NLRP3 levels compared to the model group. The best results were seen in the 80 mg/kg RA treatment group. These findings suggest that RA has a beneficial effect in treating oral ulcers [144]. *Salvia sclarea* L. ethanolic extract, containing rosmarinic acid as the most abundant constituent, was tested on 30 Wistar rats with lipopolysaccharide (LPS)-induced periodontitis to evaluate its suppressive effect on inflammation. The rats were divided into five groups: Groups 1 and 2 served as controls (Group 1 received saline injection and was treated with distilled water, and Group 2 was injected with saline and treated with *S. sclarea* extract), Group 3 (injected with LPS and treated with distilled water), Group 4 (injected with LPS and treated with *S. sclarea* extract), and Group 5 (injected with LPS and treated with *S. sclarea* extract 3 days prior to the injection and throughout the examination). The treatment was administered twice daily for 10 days. HPLC analysis showed that RA was the most abundant compound (165.30 ± 0.60 µg/mg) compared to caffeic acid (0.95 ± 0.03 µg/mg), luteolin (0.50 ± 0.02 µg/mg), apigenin (0.22 ± 0.01 µg/mg), luteolin-7-O-glucoside (5.55 ± 0.46 µg/mg), and apigenin-7-O-glucoside (8.51 ± 0.80 µg/mg). The control groups showed no inflammation or bone destruction, and there were no noticeable differences in the number of cytokines after 10 days. Groups 4 and 5 exhibited an increase in the number of fibroblasts and significantly decreased levels of TNF-α, IL-1β, and IL-6 by the end of the examination. However, the *S. sclarea* extract administered before the LPS injection did not have significant preventive effects, as it only significantly reduced IL-1β levels compared to Group 4, which started treatment after periodontitis induction. The positive effects were mainly attributed to RA content, but further clinical studies are needed to establish its role in this disease [145].

### 2.5. Eugenol (EUG)

#### 2.5.1. History

It was first isolated as a volatile compound from Eugenia caryophyllata (Myrtaceae family) back in 1929 [146]. It has a long history of use in dentistry, but it gained popularity with the zinc-oxide paste formulation, which was used in several endodontic sealers [147].

#### 2.5.2. Chemical Structure

**Figure 5:**
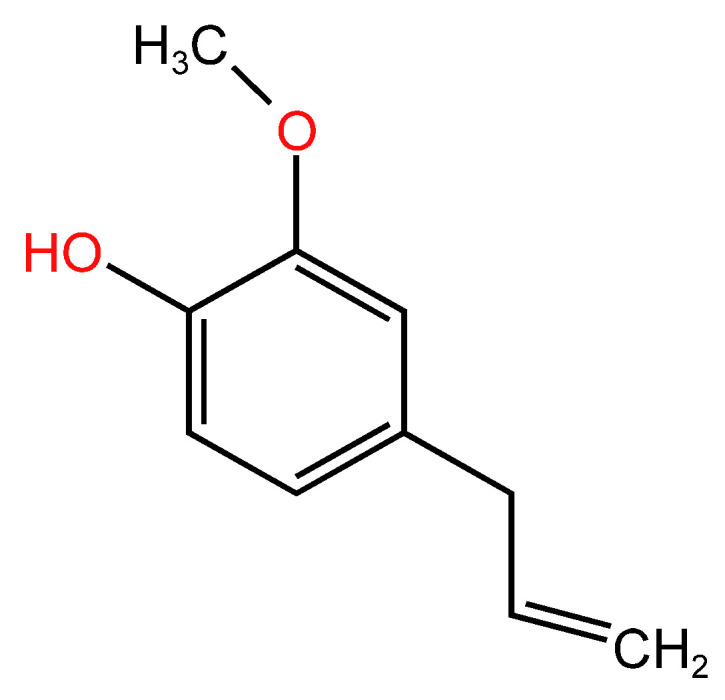
Molecular structure of eugenol.

Eugenol (Figure 5) is a natural volatile compound that belongs to the phenylpropainoid class. The allylic, hydroxyllic, and aromatic groups are the molecule’s active sites. The clove plant (Eugenya caryophyllata) is the primary source of eugenol extraction, but several other plants (cinnamon, nutmeg, and basil) also contain it [148,149].

#### 2.5.3. Antimicrobial Spectrum and Mechanism of Action

It is a great antiseptic that acts against both gram-positive and gram-negative bacteria, fungi, viruses, and several parasites [146,150].

The hydroxyl groups from EUG alter the cytoplasmic membrane structure by interacting with the bacterial cellular proteins. This interaction causes the rupture of the membrane, causing the leakage of the intracytoplasmic components. Another mechanism underlying the antibacterial effect is mitochondrial dysfunction through inhibition of the ATPase and the release of ROS, which can cause DNA damage and lead to cell death [146,151].

The antifungal effect was observed to be linked to the binding of ergosterol, which is a crucial component of the fungal membrane that is involved in permeability and fluidity regulation. Other mechanisms are similar to the antibacterial ones [152,153]. EUG prevents viral replication, but the mechanism of action is not yet fully understood [146,154].

#### 2.5.4. Formulations and Current Use of Eugenol in Dentistry

It is found in several formulations destined for oral use, such as mouthrinses, toothpaste, chewing lozenges, gels, sprays, pastes, and dental cement [155,156,157].

Eugenol has many benefits for dental and oral care due to its antimicrobial activity. It is effective against several pathogens related to dental caries. Its antioxidant, anti-inflammatory, and analgesic properties make it a useful alternative for alleviating dental pain. Several studies have implied its beneficial role in treating periodontal diseases because it inhibits pro-inflammatory mediators such as prostaglandins, several interleukins, COX-1, COX-2, leukotrienes, tumor necrosis factor-alpha, nuclear factor kappa b, and 5-lipoxygenase, reducing pulp swelling and local pain [146,150,158]. It is a traditional cement that remains in use to this day for root canal sealers [159]. Its effectiveness against *Candida* spp. and certain viruses, including HSV-1 and HSV-2, has been previously reported, indicating its potential to combat yeast and viral infections [159,160,161]. In a study, EUG demonstrated a positive action against oral cancer, showing a dose-dependent effect on the cancerous cell culture. Additionally, newer delivery systems, such as nanobio-conjugates, can improve the delivery of the compound and achieve better results [162]. Evidence supporting eugenol usage is presented in Table 5.

#### 2.5.5. Adverse Effects

Eugenol is considered to be generally safe. However, there have been reports of allergic contact dermatitis, gingivitis, minimal oral irritation, and a burning sensation in the mouth following oral use [166,167,168,169].

#### 2.5.6. Experimental Methods Used to Evaluate the Efficacy and Safety of EUG in Dental Applications

A study evaluated eugenol’s cytotoxic effect on oropharyngeal squamous cancer and osteosarcoma cells. The MTT assay revealed that EUG has a dose-dependent cytotoxic effect on both cancerous cell lines (SAOS-2 and Detroit-562), reducing the viability of the cells by 10% at a 0.1 mM concentration, and by 90% at a 1 mM concentration after 72 h. Cellular morphology was assessed by microscopic evaluation, and shrinkage in the SAOS-s cells, a more rounded appearance, and detachment were observed after 72 h. The most evident changes were observed at 1 mM, although alterations in cell morphology were seen at concentrations from 0.5–1 mM concentrations. Detroit-562 cells were treated to a concentration ranging from 0.5 to 1 mM of EUG and changes were noticed to occur in a concentration-dependent manner, but although the cells suffered shrinkage, they were not detached. Afterward, DAPI staining was performed to analyze changes in the nuclei morphology after 72 h treatment. Visible changes were observed in both cancerous cell lines exposed to 0.5 and 1 mM concentrations; the round shape of the nuclei before treatment changed from round to a fragmented, condensed shape, and their circumference area was decreased post-treatment. Apoptosis occurred even at a 0.5 mM concentration after 72 h exposure. To further investigate EUG’s ability to induce apoptosis at 0.5 mM, the Caspase glo kit was employed to measure caspase (3/7, 8, 9) activity, and RT-PCR was performed to observe the apoptosis-related gene expression (Bcl-2-an anti-apoptotic marker; Bax and Bad-pro-apoptotic markers). After 72 h, a concentration of 0.5 successfully increased caspase activity, Bax, and Bad and decreased Bcl-2 expression in both cancerous cell lines, suggesting its pro-apoptotic effect. Its ability to induce apoptosis was confirmed after Real-Time Glo annexin V apoptosis and necrosis assay. After treatment with 0.05 mM eugenol, destruction in the membrane’s structure was observed in a time-dependent manner, an indicator of necrosis after treatment with 0.5 mM eugenol, showing its beneficial action against oropharyngeal squamous cancer and osteosarcoma cells. The same study was also conducted in ovo, using HEM-CAT assay to evaluate eugenol’s potential toxicity and irritative effect. The 1 mM concentration was used for 5 min, and lysis and coagulation at the vascular level were observed, although a lower level of irritation was described. These studies showed that EUG could serve as a potential treatment for osteosarcoma and oropharyngeal cancer, with lower toxicity than conventional cancer treatment [170].

In a study conducted on 270 patients, the effects of EUG-based paste, CHX 0.2%, and the control group were compared in the treatment of alveolar osteitis after 3rd molar extraction. Postoperative pain, inflammation, infection, and wound healing were evaluated. Treatment was applied once, and the results were analyzed after 1 week. Additionally, patients were administered metronidazole (×3/daily for 3 days) and a combination of aceclophenac + seratipeptidase (×2/daily for 3 days) to measure the criteria for alveolar osteitis (if 2 of 3 criteria are met, that means the inflammation did not reduce: throbbing pain between days 3–5 that is not alleviated by analgesics, resorbed blood clot dark fragments, and painful extraction). The visual analog scale was used to assess postoperative pain, and the periodontal probe was used to measure epithelialization. Both the EUG and CHX groups significantly reduced the pain score 1, 3, and 7 days post-treatment, with the EUG group showing the best results. Additionally, in the EUG group, no incidence of osteitis after 7 days was reported, unlike in the CHX group, where 2 cases of osteitis occurred. Wound healing and inflammation were also better in the EUG group after 7 days. Overall, EUG was superior at alleviating pain, reducing inflammation, promoting wound healing, and reducing the incidence of the disease [171]. A clinical trial was performed on 100 patients to test the efficacy of eugenol in relieving pain associated with emergency coronal pulpotomies in teeth with irreversible pulpitis. Initially, the trial included 100 subjects, but several factors (inability to reach the subjects, misdiagnosis, and low initial pain score) resulted in only 78 patients remaining for examination. The subjects were divided into two groups: Group 1 (40 subjects) received one drop of pure eugenol (EUG) on a cotton pellet covered by a temporary filling material without eugenol, and Group 2 (38 subjects) received one drop of articaine from a 40 mg/mL anesthetic cartridge with 1/200,000 adrenaline. The outcomes were examined after 1, 3, and 7 days using the 0 to 10 numeric rating scale for pain. On Day 1, the EUG group showed a greater reduction in pain compared to the articaine group. Over the 7-day period, pain gradually decreased among all patients, with 70.5% experiencing no pain and almost 90% having a pain score of 3 or below at the end of the trial. EUG performed better overall as it maintained a slightly lower pain level due to its high potency on Day 1 [172]. A nano-zinc oxide EUG (NZOE) sealer was tested on 60 patients with irreversible pulpitis to determine its effectiveness in reducing the incidence of flare-ups in mandibular first molars. The patients were divided into two equal groups: Group 1 received NZOE sealers, and Group 2 used AH-26 sealers. The results were examined using the visual analogue scale 6, 18, 24, and 48 h post-treatment. After 24 h NZOE group experienced significantly lower pain compared to the other group. The trial ended after 48 h because both groups reported no swelling [173]. An oxide eugenol paste was tested on patients with dry sockets to evaluate its potential benefit in alleviating the pain related to this condition. In the trial, 90 subjects were included and divided into three groups: Group 1 (30 subjects)—honey treatment, Group 2 (30 subjects)—zinc oxide EUG paste (placed into the socket), and Group 3 (29 subjects, as one patient lost to follow-up)—normal saline rinse (control). Patients’ pain scores were evaluated post-treatment using the visual analogue scale (0—no pain; 1–3—mild pain; 4–6—moderate pain; 7–10—severe pain) at intervals of 5 min, 30 min, 60 min, 2 days, and 4 days. Subjects were allowed to take ibuprofen (200, 400, or 600 mg twice daily) based on their pain level. After 5 min of treatment, Groups 1 and 2 experienced moderate pain, which reduced to mild pain after 30 min. By Day 2, the patients’ pain score was mild, and they administered 200 mg ibuprofen. After 4 days, all groups were pain-free. The EUG group showed the best results in relieving pain and discomfort in patients with dry sockets, as the pain gradually decreased from 5 min post-treatment until the 2nd day, when the pain score reached 0 [174]. Alhamshary et al. investigated the effect of a chitosan-zinc oxide eugenol mix in pulp capping on 36 badly decayed primary molars in patients aged 4 to 8 years who were indicated for pulpotomy. Patients were divided into two equal groups: Group 1 received a chitosan-zinc oxide eugenol mix as pulp capping material, while Group 2 received zinc oxide eugenol after formocresol application. The results were examined clinically and radiographically. After the 3-month trial, the best results were observed in the chitosan-zinc oxide eugenol group, with an almost 95% success rate (17 out of 18 teeth showed no signs of inflammation) compared to the zinc oxide eugenol group, which had an approximately 84% success rate [175]. Another study was conducted on 36 primary molars with pulp necrosis from children between 3 and 8 years old. At the end of the 36-month trial, the best outcome was observed in the pulpotomy with zinc oxide paste group, which had a clinical success rate of almost 91% compared to the other group treated with lesion sterilization and tissue repair with a paste with chloramphenicol, tetracyclin, zinc oxide, and EUG, which presented a success rate of 86.4% [176].

## 3. Conclusions

Natural compounds remain a viable alternative to conventional treatment due to their broad spectrum of action against a variety of pathogens responsible for oral problems. They often prove to have an equally beneficial effect as synthetic compounds. A major factor that makes people opt for plant-based products is their lower incidence of unpleasant outcomes, which can be an especially important trait for people suffering from debilitating diseases such as oral cancer. To acknowledge the potential occurrence and intensity of the adverse effects, several experimental methods and assays were discovered and remain, to this day, standard practice. These tools provide meaningful information about a compound’s safety profile, efficacy, and mechanism of action through which they occur, making them essential in the development of safer therapeutic compounds with a higher potency and faster effect.

## Figures and Tables

**Table 1:** Supported evidence of curcumin usage.

Uses	Formulation	Type of Study	Spectrum	Results	Ref.
Gingivitis	CUR mouthwash (0.06%, 0.1%, 1%, 20%)	Systematic review	-	Curcumin mouthwash is a safer alternative than CHX with fewer side effects in the management of plaque reduction and gingivitis.	[36]
Chronic gingivitis	CUR toothpaste	Randomized controlled trial	-	This formulation successfully reduced dental plaque and gingival index even after 2 weeks of use.	[37]
Dental caries	CUR solution	In vitro	*S. mutans*	Curcumin presented a bacteriostatic effect at 125 µmol/L and reduced biofilm mass at a concentration of 15 µmol/L. These findings indicate its beneficial effect on dental caries prevention.	[38]
Periodontitis	CUR gel	Clinical trial	-	Treatment with scaling and root planing, followed by application of the gel containing 10 mg of curcumin, was administered for 5 min, once daily, for 1 month, resulting in a decrease in IL-1β and TNF-α levels. Additionally, improvements in Zn, Mg, and Cu levels were observed, making curcumin a promising candidate for periodontitis treatment.	[39]
Oral candidiasis	Topic CUR nanoparticles (NP)	In vivo	*C. albicans*	Treatment with CUR NP was followed for 10 days with 2 applications daily. It exhibited a bacteriostatic effect at 64 µg/mL. It improved oral lesions after 5 days, and by the 10th day, its effect was comparable to nystatin in rat models.	[40]
Oral mucositis (OM)	10 mg/g CUR gel	Controlled trial	-	After 2 weeks of treatment, significant improvements were observed in erythema, ulcer size, and pain levels, with noticeable results evident after just one week.	[41]
Recurrent aphthous stomatitis	1% CUR nanomicelle gel, 2% CUR gel	Randomized clinical trial	-	The treatment was administered for one week with three daily applications. Both formulations showed improvement in pain score and lesion size after one week. However, the 1% CUR nanomicelle gel was more effective, showing visible results even after 4 days.	[19]

**Table 2:** Supported evidence of quercetin usage. The symbol “↓” indicates “reduced”.

Uses	Formulation	Type of Study	Spectrum	Results	Ref.
Dental caries	-	In vitro	*S. mutans*	It ↓ biofilm formation even more than CHX. It presented bactericidal action against *S. mutans* comparable to CHX, making it a safer alternative in dental caries prevention.	[73]
Periodontal disease	QRC nanoemulsion	In vitro	*T. forsythia*, *P. gingivalis*	*P. gingivalis* was sensitive to QRC action at a concentration of 100 µg/mL and *T. forsythia* was sensitive at both 50, and 100 µg/mL. These results suggest its positive effect on the management of periodontal disease.	[62]
Oral mucositis (OM)	QRC capsules (250 mg)	Randomized double-blind clinical trial	-	QRC ↓ the relative risk of OM in patients following chemotherapy treatment. It has proven to be a suitable adjuvant treatment with no side effects in this debilitating condition.	[74]
Oral infections	QRC mouthwash at 25, 50, 100 µL	In vitro	*C. albicans*, *E. faecalis*, *S. aureus*, *S. mutans*, *Lactobacillus*	QRC presented antimicrobial effects in a dose-dependent manner, making it useful in the treatment of various pathogen-related oral infections.	[65]

**Table 3:** Supported evidence of allicin usage.

Uses	Formulation	Type of Study	Spectrum	Results	Ref.
Dental caries	-	In vitro	*S. mutans*, *S. sobrinus*, *A. oris*	At a concentration of 600 µg/mL, allicin exhibited a bacteriostatic effect against *S. mutans*. Following 1-h exposure at concentrations of 2200, 4500, and 9800 µg/mL, it demonstrated a dose-dependent bactericidal effect on *S. mutans* biofilms. However, it did not destroy the biofilm structure. While the findings suggest potential benefits for caries prevention, further studies are necessary to validate its efficacy against this disease.	[107]
Periodontitis	-	In vitro	*F. nucleatum*, *A. actinomycetemcomitans*	The bacteriostatic effect was exhibited at a concentration of 300 µg/mL, making it a potential treatment for periodontitis.	[107]
Chronic periodontal disease	-	In vitro	*P. gingivalis*, *P. gingivalis* deficient mutant	A bacteriostatic effect was observed at 2400 µg/mL. It successfully inhibited the mutant strain at a concentration below 300 µg/mL, indicating that it can have a protecting action against periodontium destruction.	[107]
Stage II oral submucous fibrosis	1% allicin injection	Randomized clinical trial	-	After the treatment, there was an improvement in overall oral health. It alleviated the burning sensation and helped with the mouth opening.	[99]
Recurrent aphthous stomatitis	Allicin mouthrinse, capsules	Clinical trial	-	Both formulations significantly reduced ulcer size within 7 days of treatment. However, allicin is not effective in preventing recurrence.	[100]

**Table 4:** Supported evidence of RA usage.

Uses	Formulation	Type of Study	Spectrum	Results	Ref.
Oral candidiasis	RA solution	In vitro	*C. albicans*, *C. krusei*, *C. glabrata*, *C. tropicalis*, *C. parapsilosis*	It exhibited antifungal activity at 0.1–0.2 mg, making it a potential alternative in the treatment of yeast infections.	[117]
Biofilm reduction	200 mg/mL RA solution	In vitro	*S. aureus*, *P. aeruginoa*, *C. albicans*, *E. faecalis*, *S. mutans* and *C. albicans*	It reduced the viability of all pathogens, suggesting its beneficial role in preventing biofilm formation that can lead to oropharyngeal problems.	[131]
Oral cancer	-	In vitro	-	It was observed that RA has a dose-dependent effect on oral cancerous cells, presenting the highest activity at 40 µM.	[132]

**Table 5:** Supported evidence of EUG usage. The symbols “↓” and “↑” indicate “reduced” and “increased”.

Uses	Formulation	Type of Study	Spectrum	Results	Ref.
Biofilm reduction	-	In vitro	*C. albicans*, *S. mutans*	At a concentration of 100 µg/mL EUG inhibited single and mixed biofilm by approximately 30% for the single strains, and by 50% in mixed biofilm. At 200 µg/mL, it significantly ↓ the viability of single and mixed biofilms.	[160]
Dental caries development	EUG nanoemulsion	In vitro	*S. mutans*	16 mg/mL EUG ↓ total flora counts after 5 weeks in rat models, ↓ the incidence of smooth surface, and sulcal caries.	[163]
		In vivo	*S. mutans*	EUG at concentrations of 4, 8, and 16 mg/mL ↓ acid production, bacterial adherence, and suppressed water-insoluble glucans synthesis in a dose-dependent manner in rat models, making it a potential candidate for dental caries prevention.	[163]
Periodontitis	-	In vitro	*P. gingivalis*	EUG exhibits bacteriostatic activity at 31.25 µM with a significant decrease in cell viability after 4 h, and a bactericidal effect at 125 µM. At 15.6 µM it ↓ biofilm formation by 90% and down-regulated virulent factor genes of *P. gingivalis.* Additionally, it has the ability to ↓ already-formed biofilm by 40% at 125 µm. These results suggest that eugenol could be a safer alternative for periodontitis treatment.	[164]
Tongue squamous cell carcinoma	-	In vitro	-	After 72 h of exposure to different concentrations of EUG (0.1, 0.25, 0.5, 0.75, 1 mM), a dose-dependent decrease in cell viability was observed. A concentration of 0.5 mM significantly ↑ expression of pro-apoptotic mRNA markers (Bax, Bad), indicating eugenol’s antitumor effect.	[165]

## Data Availability

The original contributions presented in the current study are included in the article. Further inquiries can be directed to the corresponding author.
